# Asymmetric Bilateral Hip Dislocations With an Associated Unstable Pelvic Ring Injury: A Case Report

**DOI:** 10.7759/cureus.27344

**Published:** 2022-07-27

**Authors:** Jacob Shermetaro, Jourdan Gard, Kelley Brossy

**Affiliations:** 1 Orthopedic Surgery, Beaumont Health, Farmington Hills, USA

**Keywords:** internal iliac artery, internal pudendal artery, unstable pelvic ring injury, pelvic ring injury, femoral head fracture, femoral head fracture-dislocation, posterior hip dislocation, anterior hip dislocation, hip dislocations, bilateral hip dislocation

## Abstract

Asymmetric bilateral hip dislocations are unusually rare injuries in isolation; however, they are even less often associated with unstable pelvic ring injuries. We report a case of asymmetric bilateral hip dislocations with an associated unstable pelvic ring injury and femoral head fracture in a 46-year-old male after being struck by a vehicle as a pedestrian. These injury patterns are typically the result of high-energy trauma, often seen in motor vehicle accidents. A thorough trauma evaluation, including proper radiographic and clinical evaluations, is necessary for the workup of these patients. This case presents an unusual combination of injuries that are associated with severe, potentially life-threatening complications. Ensuring a timely diagnosis and the initiation of early, proper management are essential in preventing poor outcomes in these patients.

## Introduction

Asymmetric bilateral hip dislocations are unusually rare injuries described as one hip dislocating posteriorly while the contralateral hip dislocating anteriorly. These injuries account for approximately 0.01-0.02% of all joint dislocations [1]. There have been around 100 total reported cases in the literature, with only 33 cases specifically reported in the English literature as of 2018 [2]. Even fewer of these injuries have been described with an associated unstable pelvic ring injury. We report a case of asymmetric bilateral hip dislocations with an associated unstable pelvic ring injury and femoral head fracture.

## Case presentation

**Figure 1:**
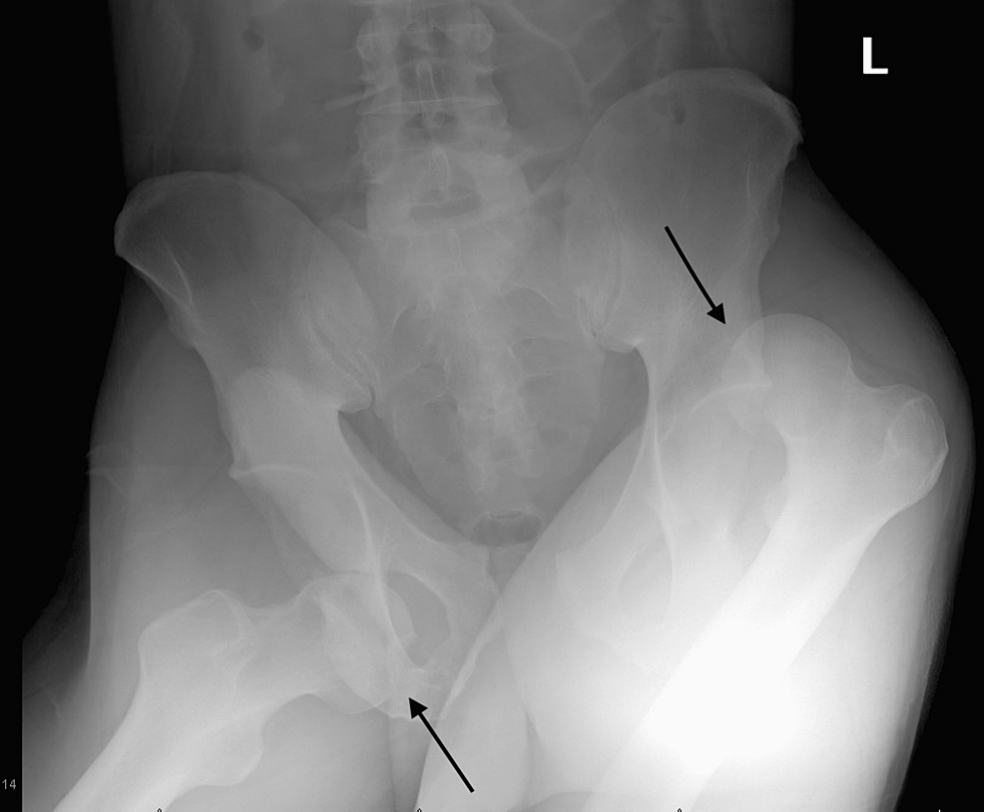
Initial pelvis radiograph demonstrating left posterior superior (right arrow) and right anterior inferior (left arrow) native hip dislocations with pubic diastasis, right sacroiliac joint widening and left sacral ala fracture with distal extension

A 46-year-old male with no significant past medical history presented to the emergency department with bilateral hip and leg pain. He was intoxicated upon arrival and provided a limited history of the events preceding his injury. He was reportedly a pedestrian that was struck and run over by a vehicle. Physical examination in the emergency department showed the right leg externally rotated, abducted, and flexed at the hip. The left leg was flexed at the hip, adducted, and internally rotated. There were no signs or symptoms of compartment syndrome and no obvious neurovascular injuries noted at this time. Radiographs demonstrated left posterior superior and right anterior inferior native hip dislocations with pubic diastasis (excessive widening of the pubic symphysis), right sacroiliac joint widening, and a left sacral ala fracture with distal extension (Figure 1).

**Figure 2:**
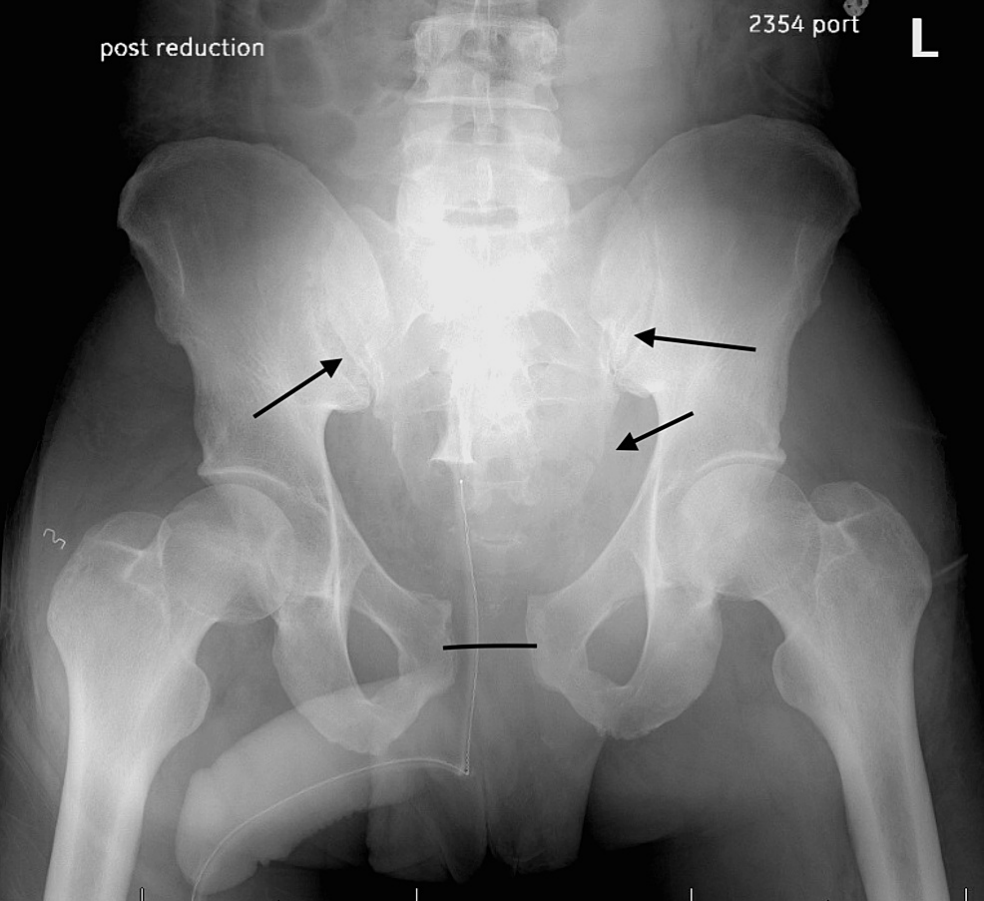
Post-reduction pelvis radiograph demonstrating bilateral concentric hip reductions and redemonstrating pubic symphysis widening (black line), left sacral fracture (two right arrows), and right sacroiliac joint widening (left arrow)

In the emergency department, bilateral hips were close reduced under conscious sedation. Following reduction, each hip was ranged and found to be stable. A post-reduction pelvis radiograph confirmed bilateral hip reduction (Figure 2).

**Figure 3:**
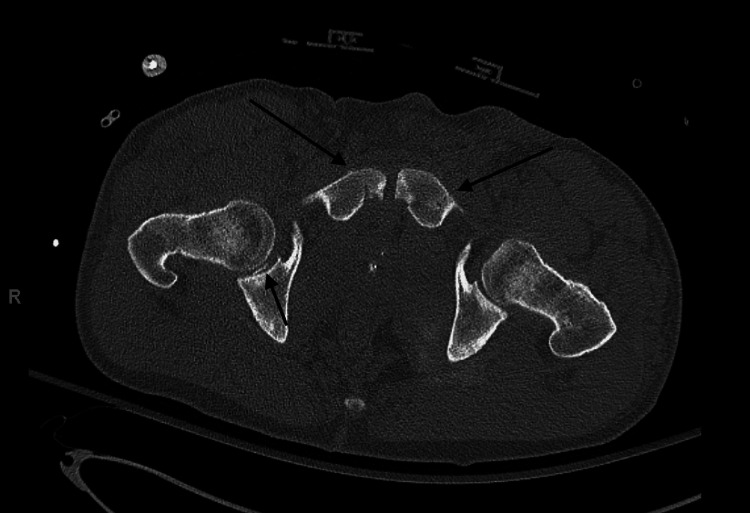
Axial view of pelvis computed tomography scan demonstrating bilateral non-displaced superior pubic rami fractures and an intraarticular bony fragment in the right hip joint

**Figure 4:**
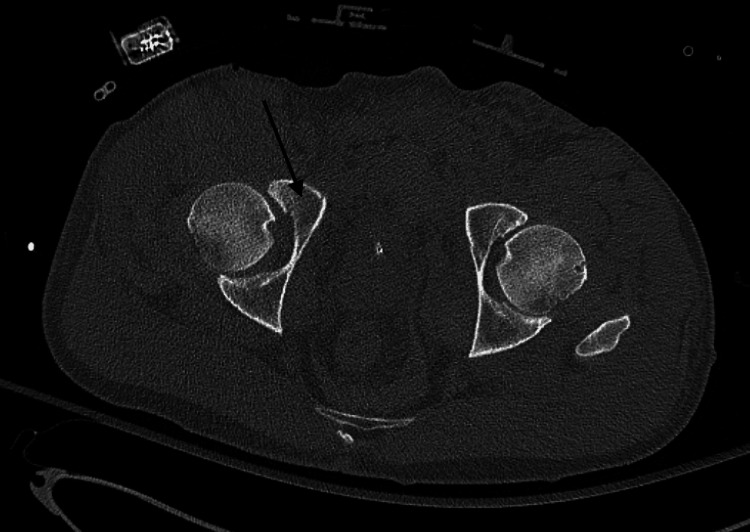
Axial view of the pelvis computed tomography scan demonstrating a non-displaced anterior wall fracture of the right acetabulum

Next, a computed tomography angiography (CTA) of the abdomen and pelvis was obtained and revealed bilateral nondisplaced superior pubic rami fractures, a right non-displaced anterior wall acetabulum fracture, and posterior impaction of the right femoral head with intra-articular fragments in the right hip joint (Figures 3-4).

**Figure 5:**
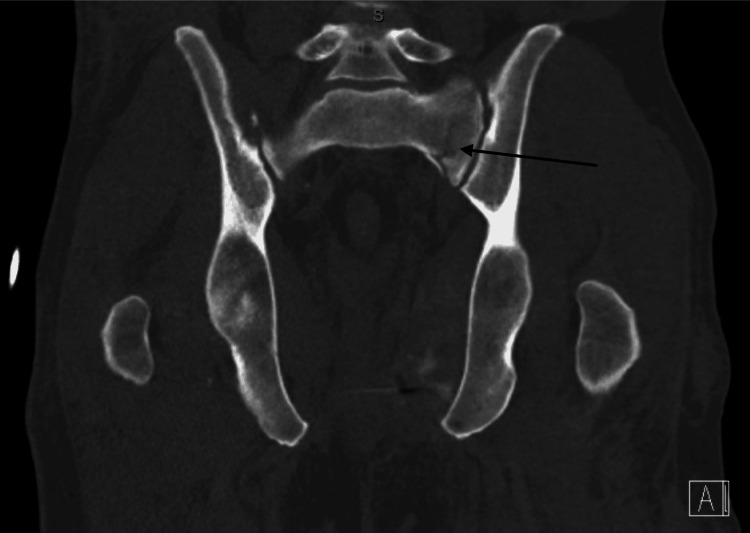
Coronal view of the pelvis computed tomography scan demonstrating non-displaced left sacral ala fracture

**Figure 6:**
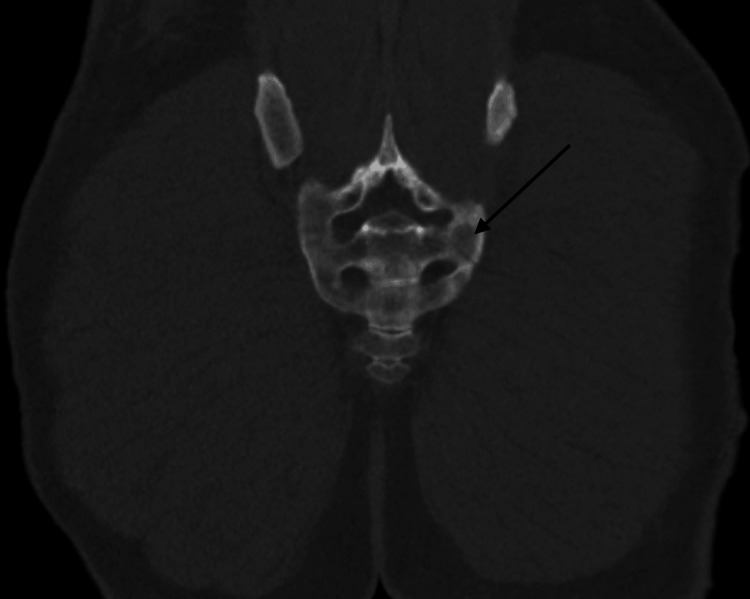
Coronal view of the pelvis computed tomography scan demonstrating distal extension of the non-displaced left sacral fracture

The left-sided sacral fracture with distal extension was re-demonstrated (Figures 5-6).

**Figure 7:**
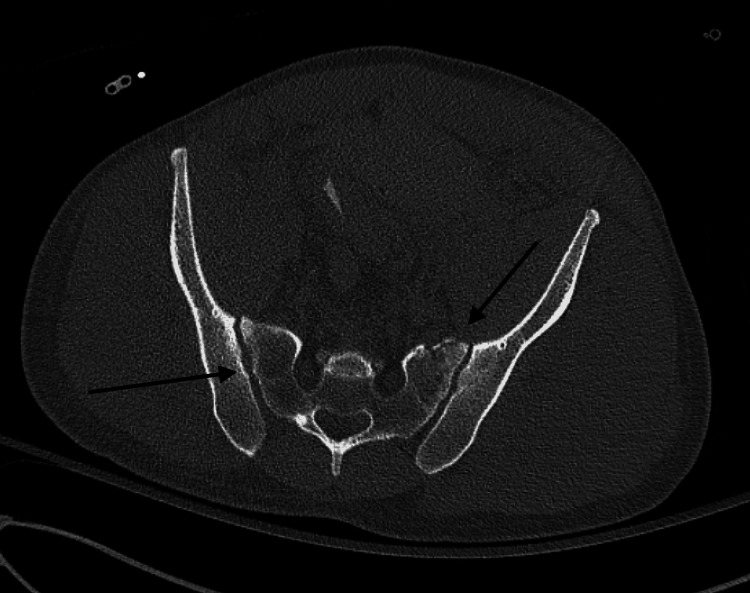
Axial view of the pelvis computed tomography scan demonstrating no widening at the right sacroiliac joint with left-sided sacral ala fracture re-demonstrated

The right sacroiliac joint widening is no longer seen here as a pelvic binder had been placed prior to CTA given pubic symphysis widening seen on the pelvis radiograph (Figure 7).

Additional bony injuries included bilateral nondisplaced 1, 2, 3, and 4 lumbar transverse process fractures. A right distal femoral traction pin was placed to take pressure off the incarcerated fragment of bone in the right hip joint. It was noted that computed tomography angiography also revealed a pelvic hematoma with active extravasation, indicating vascular injury. Shortly after traction pin placement, the patient began to exhibit signs of hemodynamic instability despite pelvic binder placement and resuscitation efforts. The patient was taken emergently to the interventional radiology suite for embolization of his left internal pudendal and left internal iliac arteries.

**Figure 8:**
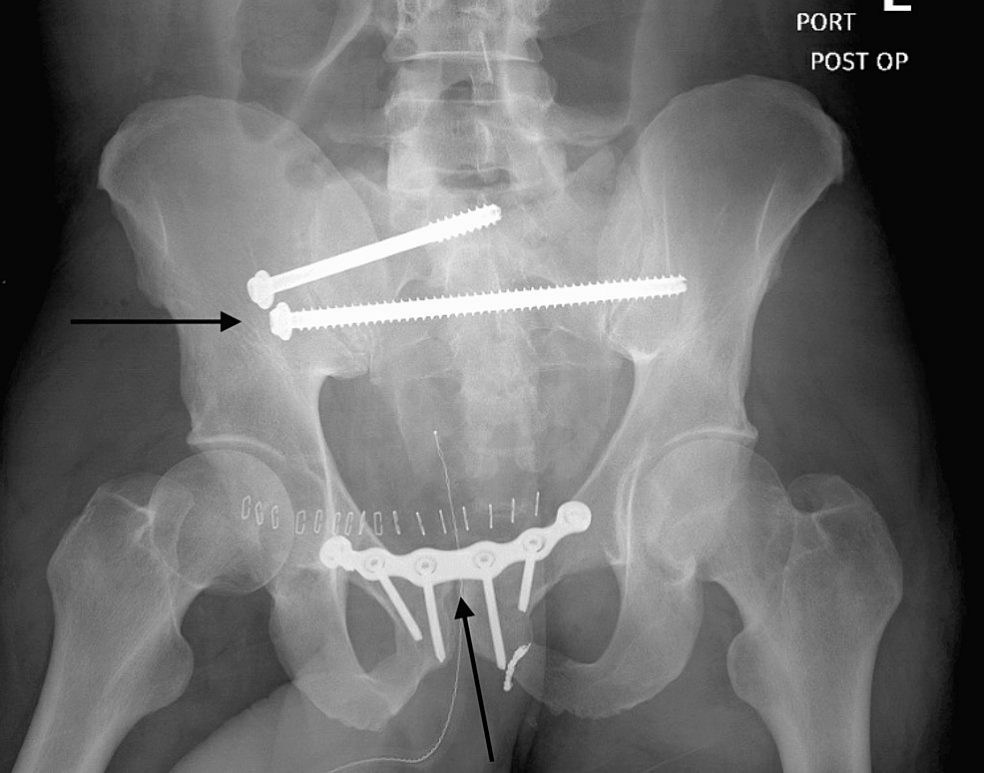
Anterior-posterior pelvis post-operative radiograph demonstrating interval hardware placement and reduction of right sacroiliac joint and pubic symphysis

**Figure 9:**
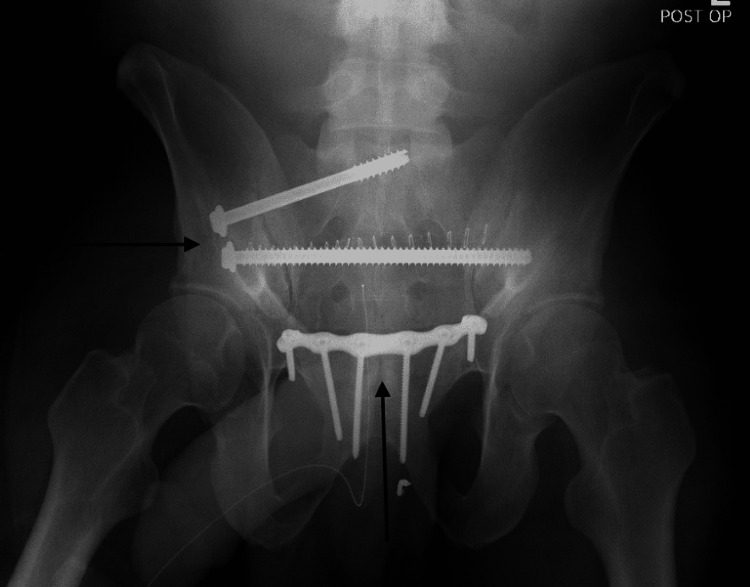
Anterior-posterior outlet view, pelvis post-operative radiograph demonstrating interval hardware placement and reduction of right sacroiliac joint and pubic symphysis

**Figure 10:**
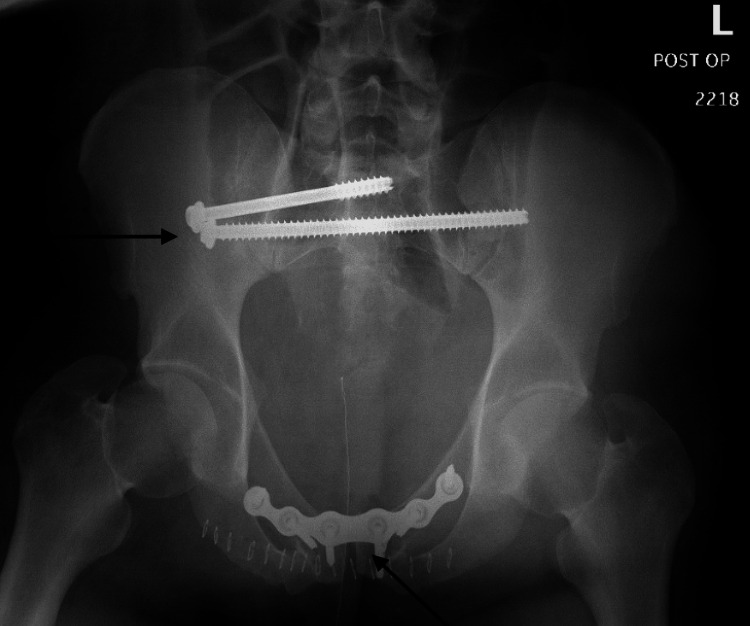
Anterior-posterior inlet view, pelvis post-operative radiograph demonstrating interval hardware placement and reduction of right sacroiliac joint and pubic symphysis

Once stable the following day, he was taken to the operating room for open reduction internal fixation of the pubic symphysis and percutaneous sacroiliac screws (Figures 8-10).

He returned to the operating room two days later for an arthrotomy of the right hip with the removal of incarcerated bony fragments. He was instructed to be non-weight-bearing on his right lower extremity and toe-touch weight-bearing on his left lower extremity. He was discharged from the hospital to a rehabilitation facility in stable condition several days later. At his three-month follow-up appointment, his radiographs demonstrated all fractures to be well-healed with hardware in place. He was in minimal pain, tolerating weight bearing on his bilateral lower extremities, and had returned to work.

## Discussion

Asymmetric bilateral hip dislocations were first described in 1845 following a wagon turnover accident [1]. Males are more commonly affected, and the average age of affected patients is 32.9 years [1]. It has been estimated that bilateral hip dislocations have an incidence of 1.25% of all hip dislocations, and 40% of these are asymmetric [1]. These are often high-energy injuries as the hip is an inherently stable joint and about 400 newtons of force is required to cause a hip dislocation; therefore, it is no surprise that there is a 95% incidence of injury to other areas of the body [3]. The direction of dislocation is related to the vector of force applied to the hip joint. It is also important to note that the hip position plays a large role in the injury pattern. If the hip is in abduction and dislocated anteriorly, it may not cause significant acetabular or femoral head bony fractures; however, if the hip is in adduction, the forces may result in acetabular or femoral head fractures. Posterior hip dislocations are more common than anterior. Some sources estimate that up to 90% of all native hip dislocations are posterior. This is a result of weaker bony and soft tissue support inferior and posterior to the hip joint. A posterior vector on the hip is also more commonly encountered, often with the hip in a flexed position, as seen in dashboard injuries [1].

A thorough physical examination and radiographic evaluation are required for the workup of any native hip dislocation. These injuries are typically the result of high-energy trauma, and there must be a high index of suspicion for associated injuries. Following proper Advanced Trauma Life Support management, a close inspection of radiographs should be performed. Treatment and complications of these injuries are largely dictated by initial management. Most literature advocates for urgent reduction within six hours of dislocation [4]. The incidence of avascular necrosis is approximately 2-10% of these injuries and continues to rise after six hours of dislocation [5]. Closed reduction maneuvers may differ depending on if the hip dislocation is anterior or posterior. Therefore, accurate diagnosis is important prior to a closed reduction attempt [4]. Posterior hip dislocations may present with the extremity shortened, hip flexed, internally rotated, and adducted, while anterior dislocations may present with the extremity lengthened, hip flexed, externally rotated, and abducted. In some cases, it may be difficult to assess the direction of dislocation based on anterior-posterior (AP) pelvis radiographs. Possible hints are that posterior dislocations are generally superior while anterior dislocations are generally inferior. There may also be fractures of the anterior or posterior walls of the acetabulum aiding in this assessment [1]. The AP pelvis radiographs should also be evaluated to rule out obvious femoral neck fractures to dictate if a closed reduction attempt is appropriate or not. Closed reduction attempts may lead to the displacement of these fractures and cause challenges for further management. Close neurologic examination of the sciatic nerve is also important in ensuring it is not injured, which is often seen in posterior dislocations. Repeat examination after closed reduction or if reduction seems to be impeded is needed given the possibility of entrapping the nerve in the joint during reduction [1].

After reduction is confirmed with radiographs, one should range the hip joint to assess for stability to dictate further management and the need for possible skeletal traction. Skeletal traction may also be used to remove pressure from the chondral surface of the femoral head if there are entrapped bony fragments in the hip joint. Post-reduction computed tomography scans should be obtained to look for possible eccentric reductions, occult fractures, or intra-articular fragments [1].

Bony pelvic ring injuries include fractures of the ischiopubic bones, sacroiliac joints, and sacrum and have been shown to have varying long-term outcomes. While several classification systems are used for these injury patterns, in short, surgical fixation is often required when findings of instability or significant displacement are present. There is no consensus in the literature with regards to the optimal timing of surgery, but there is a trend toward early fixation [6]. Emergent or urgent surgical intervention may be required when hemodynamic instability is present. Exsanguination is the leading cause of death in severe pelvic ring injuries, with the source of bleeding most commonly being venous in origin. This is often controlled with pelvic binders, orthopedic surgical stabilization, and pre-peritoneal pelvic packing. Arterial bleeding sources are most commonly controlled with angiography and embolization [7]. Orthopedic surgical techniques vary widely and can include external fixator placement, open reduction internal fixation, or percutaneous fixation, with many considerations for each option. For example, external fixation placement may be postponed or adjusted if abdominal surgery is also required, and percutaneous techniques may be more appropriate if there is significant skin compromise at possible incision sites [6].

The traumatic hip dislocation with associated femoral head fracture was first described in 1869 by Birkett, and the Pipkin classification is commonly used for these injuries [8]. Optimal treatment is controversial, ranging from non-operative management, surgical excision of bony fragments in the hip joint, open reduction, internal fixation, or arthroplasty. Treatment goals include obtaining an anatomic reduction with a congruent hip joint, and literature has shown that loose bony fragments in the hip joint lead to poor outcomes when they are retained [9]. Hip dislocations associated with pelvic ring injuries and femoral head fractures are uncommon but play a large role in treatment decision-making, often requiring open surgical stabilization, which may significantly change the treatment course for these patients.

## Conclusions

Asymmetric bilateral hip dislocations with associated unstable pelvic ring injuries and femoral head fractures are exceedingly rare injuries, but they are associated with severe complications. This case shows that timely diagnosis and thorough evaluation are necessary to ensure proper management is initiated. Associated injuries must be identified as they may be life-threatening and urgent reduction of hip dislocations is needed to decrease the risk of femoral head avascular necrosis. Following initial management, surgical fixation is typically necessary when unstable pelvic ring injuries are present, with urgent or emergent intervention often needed in the setting of hemodynamic instability.
